# Tick-Borne Encephalitis Virus, Kyrgyzstan

**DOI:** 10.3201/eid1705.101183

**Published:** 2011-05

**Authors:** Benjamin J. Briggs, Barry Atkinson, Donna M. Czechowski, Peter A. Larsen, Heather N. Meeks, Juan P. Carrera, Ryan M. Duplechin, Roger Hewson, Asankadyr T. Junushov, Olga N. Gavrilova, Irena Breininger, Carleton J. Phillips, Robert J. Baker, John Hay

**Affiliations:** Author affiliations: State University of New York, Buffalo, New York, USA (B.J. Briggs, D.M. Czechowski, J. Hay);; Health Protection Agency, Porton Down, Salisbury, UK (B. Atkinson, R. Hewson);; Texas Tech University, Lubbock, Texas, USA (P.A. Larsen, H.N. Meeks, J.P. Carrera, R.M. Duplechin, C.J. Phillips, R.J. Baker);; National Academy of Sciences of the Kyrgyz Republic, Bishkek, Kyrgyz Republic (A.T. Junushov);; Ministry of Healthcare of the Kyrgyz Republic, Bishkek (O.N. Gavrilova, I. Breininger)

**Keywords:** Encephalitis, Kyrgyzstan, vector-born infections, tick-borne, flavivirus, viruses, zoonoses, dispatch

## Abstract

Tick-borne encephalitis virus (TBEV) is an emerging pathogen in Europe and Asia. We investigated TBEV in Kyrgyzstan by collecting small mammals and ticks from diverse localities and analyzing them for evidence of TBEV infection. We found TBEV circulating in Kyrgyzstan much farther south and at higher altitudes than previously reported.

Tick-borne encephalitis virus (TBEV) is a flavivirus in the family *Flaviviridae*. The TBEV positive-sense RNA genome is translated as a polyprotein and subsequently cleaved into 3 structural and 7 nonstructural (NS) proteins (*1*). TBEV has 3 subtypes— European, Siberian, and Far-Eastern—each of which has its own ecology, clinical presentation, and geographic distribution (*2*). The vectors are *Ixodes ricinus* ticks for the European subtype and *I. persulcatus* ticks for the other 2 subtypes. TBEV circulates through a complex cycle involving small mammals, ticks, and large mammals (*3*); it can also be transmitted through consumption of unpasteurized milk and milk products (*4*).

Our unpublished data and that of others suggest that TBEV circulates in Kazakhstan. However, we have found no reports (in English) since 1978 of TBEV infection in the neighboring Kyrgyz Republic (Kyrgyzstan). Kyrgyzstan has extensive alpine and subalpine habitats (94% of Kyrgyzstan is >1,000 m above sea level) (*5*); the Tien Shan mountain range dominates and physiographically links Kyrgyzstan to the Himalayas and western People’s Republic of China. We conducted fieldwork in Kyrgyzstan during June–July 2007 and July–August 2009 to establish a baseline of risk for zoonotic diseases, including TBEV.

## The Study

**Figure 1:**
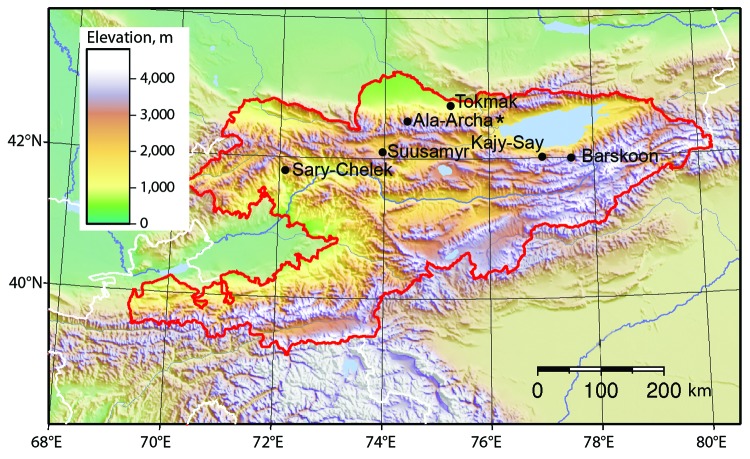
Animal trapping sites in Kyrgyzstan, with topographic characteristics shown. Ala-Archa (star) is the location of tick-borne encephalitis virus and a possible human case of tick-borne encephalitis.

During the 2007 and 2009 study periods, we collected 369 rodents and insectivores and 222 ixodid and 128 argasid ticks from 6 localities in Kyrgyzstan (Figure 1; Table 1) in accordance with animal subject review boards of Texas Tech University and the State University of New York at Buffalo. We analyzed 302 rodents and insectivores for immunoglobulin (Ig) G and IgM to TBEV by using recombinant antigen of domain III from the envelope (E) protein of Kumlinge and Powassan viruses (*6*). This assay is specific for the tick-borne flavivirus group and lacks cross-reactivity that occurs with other assays (*7*). We found that serologically positive (IgG and IgM) mammals were clustered at Ala-Archa National Nature Park, ≈40 km south of Bishkek, the capital of Kyrgyzstan, at elevations ranging from 1,891 to 2,472 m. Using mitochondrial DNA analysis, we also found clusters of seropositive Himalayan field mice, *Apodemus pallipes*.

**Table 1:** Overview of samples collected and tested for tick-borne encephalitis virus, 6 localities, Kyrgyzstan*

Animal species	No. collected			No. positive in Jun–Jul 2007/Jul–Aug 2009 (overall %)		
	Jun–Jul 2007	Jul–Aug 2009		RT-PCR†	IgG	IgM
*Alticola argentatus*	2	5		1	0	0
*Apodemus pallipes*‡	79	93		3	5/10 (8)	2/9 (6)
*Apodemus agrarius*	11	15		0	0	0/5 (19)
*Crocidura sp.*	11	2		0	0	0
*Dryomys nitedula*	11	0		0	0	0
*Microtus ilaeus*§	39	17		3	0/1 (2)	3/0 (5)
*Mictrous gregalis*	0	1		0	0	0
*Mus musculus*	3	10		0	1/1 (15)	0/2 (15)
*Myodes centralis*	1	31		0	0/2 (6)	0/2 (6)
*Rattus turkestanicus*	26	0		0	1/0 (4)	1/0 (4)
*Rattus norvegicus*	1	0		0	0	0
*Cricetulus* sp.	0	4		0	0	0/1 (25)
*Meriones* sp.	0	6		0	0	0
Total	184	185		7	7/14	6/19

*Analysis of reverse transcription–PCR (RT-PCR) and ELISA. RT-PCR data represent only 2007 samples. Ig, immunoglobulin. †5 of 7 positive samples were collected from Ala-Archa. ‡1 positive *A. pallipes* mouse was collected from Sary-Chelek. §1 positive *M. ilaeus* vole was collected from Suusamyr.

To further evaluate the prevalence of TBEV, we used reverse transcription–PCR (RT-PCR) to examine viral genomic sequences in tissue samples collected from rodents, insectivores, and ticks. We used 3 separate PCR protocols. Table 2 shows primer sequences. Real-time and conventional RT-PCRs were used; however, conventional RT-PCR was preferred because it allowed sequencing of viral genomes. Thus far, we have examined sequences from the NS5 (*8*) and E (*9*) protein coding regions.

**Table 2:** Primers used to test rodents, insectivores, and ticks for tick-borne encephalitis virus, Kyrgyzstan, 2007 and 2009*

Primer	Gene	Sequence, 5′ → 3′	Reference
FSM-1	NS5	GAGGCTGAACAACTGCACGA	(*8*)
FSM-2	NS5	GAACACGTCCATTCCTGATCT	(*8*)
FSM-1i	NS5	ACGGAACGTGACAAGGCTAG	(*8*)
FSM-2i	NS5	GCTTGTTACCATCTTTGGAG	(*8*)
TBEV913F	E	TGCACACAYYTGGAAAACAGGGA	(*9*)
TBEV1738R	E	TGGCCACTTTTCAGGTGGTACTTGGTTCC	(*9*)
RH TBE forward	E	GGCAGCATTGTGACCTGTGT	R. Hewson, unpub. data
RH TBE reverse	E	CGTGTCCTGTGGCTTTCTTTTT	R. Hewson, unpub. data
RH TBE probe	E	6FAM-AGGYGKCYTGTGAGGC-MGB NFQ	R. Hewson, unpub. data

*TBEV, tick-borne encephalitis virus; NS, nonstructural protein; E, envelope.

**Figure 2:**
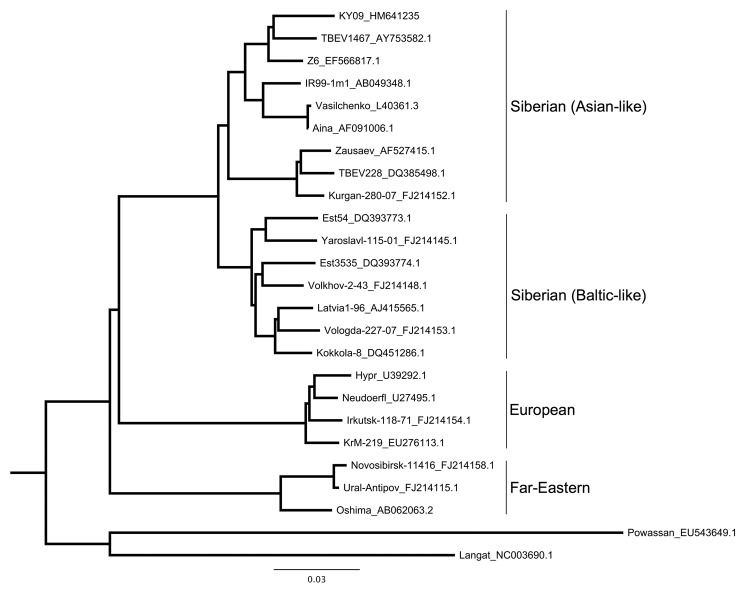
Maximum-likelihood phylogenetic tree of relationship between various tick-borne encephalitis virus (TBEV) strains isolated from rodents, insectivores, and ticks, Kyrgyzstan, 2007 and 2009. Tree is based on partial sequencing of the envelope protein (from Cys3 to Gly286). Strain names are followed by GenBank accession numbers. The strain from Ala-Archa (KY09_HM641235) is most closely related to strains from Novosibirsk (TBEV 1467 and Z6). This strain was isolated from an *Ixodes persulcatus* tick pool, representative of 5 other positive tick pools, and from liver samples from 2 *Apodemus pallipes* mice (sequence analysis of other samples not shown). Scale bar indicates nucleotide substitutions per site.

On the basis of data obtained in 2007, we focused collections in 2009 at 2 sites at Ala-Archa, 5 km apart and differing in elevation by 100 m. We found TBEV-positive ticks and IgG- and IgM-positive *A. pallipes* mice at collection sites. Sequence analyses of TBEV NS5 and E genes from *A. pallipes* mice and *I. persulcatus* ticks suggested that the TBEV circulating in Kyrgyzstan is of the Siberian subtype. Phylogenetic analyses of the E protein, amplified from a pool of *I. persulcatus* ticks collected at Ala-Archa, showed that the TBEV strain from Kyrgyzstan shares a clade with 2 strains (TBEV 1467 and Z6) isolated in the Novosibirsk region of Russia (Figure 2). This sequence is homologous with that of 5 other TBEV-positive tick pools and liver samples from *A. pallipes* mice from the same collection site.

While we were conducting fieldwork in 2009, a possible case of encephalitis occurred in a human. The 21-year-old man had removed an engorged tick (*I. persulcatus*) from himself after visiting Ala-Archa in June 2009. After ≈22 days, he sought care at the National Center for Infection in Bishkek for signs and symptoms consistent with viral encephalitis; he died 15 days later. We obtained 2 samples of the patient’s serum (in accordance with approved human Institutional Review Board protocols from the State University of New York at Buffalo) at 22 and 37 days postexposure. The TBEV IgG titer for the 22-day sample was 200, within reference range, but the titer at 37 days was 2,000. This rising titer strongly indicates infection with TBEV.

## Conclusions

Identification of the Ala-Archa National Nature Park as a focus of TBEV transmission is noteworthy because of its proximity to the capital. This TBEV focus is unlikely to be transient because we found evidence of TBEV in small mammals and ticks in 2007 and in 2009. We also found serologic evidence of human infection in 2009. Our findings are relevant to public health because Ala-Archa is frequently visited by hikers and climbers from many parts of the world. In 2008, nearly 45,000 persons visited Ala-Archa.

A TBEV focus at 2,100 m on the north slope of the Tien Shan mountains is relevant for several reasons. One of these is the fact that the east–west Tien Shan mountain range marks the approximate southernmost distribution of *I. persulcatus* ticks*,* the vectors of the Siberian and Far-Eastern strains of TBEV (*10*). Likewise, the north slope of this mountain range marks the northernmost distribution of the likely reservoir species in Kyrgyzstan, *A. pallipes* mice. Our analysis of cytochrome b DNA sequences from these mice in Kyrgyzstan supports the hypothesis that they are recent, Late Pleistocene or Holocene epoch (<15,000 years), arrivals in the region. Haplotype divergence across all collecting localities is <1%. A tentative explanation for the TBEV focus on the northern slope at Ala-Archa is that this slope is the approximate geographic point of overlap for the distribution of *I. persulcatus* ticks and a suitable reservoir species, *A. pallipes* mice. Although *A. pallipes* mice have not previously been identified as a TBEV reservoir, other *Apodemus* spp. mice in Europe and Siberia are TBEV reservoirs. Laboratory studies have shown that, in contrast with other rodents, mice of the genus *Apodemus* are capable of vertical transmission (*11*) and nonviremic transmission of TBEV through ixodid ticks (*12*).

Finding TBEV-infected ticks active at these altitudes is probably not the result of climate change. Rather, we propose altitude compensation at southern latitudes as an explanation. By altitude compensation, we mean that the closer one gets to the equator, the higher the altitude that is needed for ideal transmission ecology. We suggest that TBEV transmission in Kyrgyzstan is a delicate interaction between tick larvae, tick nymphs, and reservoir rodents, analogous to the situation seen with *I. ricinus* ticks in central Europe (*13*).

Our findings provide testable hypotheses about the ecologic and physiographic factors that determine the distribution of TBEV in Kyrgyzstan. Additional understanding of these factors will aid public health responses to the zoonosis caused by this virus (*14*).

## References

[1] Lindenbach BD, Thiel H-J, Rice CM. Flaviviridae: the viruses and their replication. In: Knipe DM, Howley PM, editors. Fields’ virology. 5th ed. Philadelphia: Wolters Kluwer Health/Lippincott Williams & Wilkins; 2007. p. 1101–52.

[2] Randolph SE. Tick-borne encephalitis virus, ticks and humans: short-term and long-term dynamics. Curr Opin Infect Dis. 2008;21:462–7. 10.1097/QCO.0b013e32830ce74b 18725794

[3] Lindquist L, Vapalahti O. Tick-borne encephalitis. Lancet. 2008;371:1861–71. 10.1016/S0140-6736(08)60800-4 18514730

[4] Holzmann H, Aberle SW, Stiasny K, Werner P, Mischak A, Zainer B, Tick-borne encephalitis from eating goat cheese in a mountain region of Austria. Emerg Infect Dis. 2009;15:1671–3. 19861072 10.3201/eid1510.090743 PMC2866415

[5] Kyrgyzstan CIA. The world factbook; 2009 [cited 2009 Dec 27]. https://www.cia.gov/library/publications/the-world-factbook/geos/kg.html

[6] Holbrook MR, Shope RE, Barrett AD. Use of recombinant E protein domain III-based enzyme-linked immunosorbent assays for differentiation of tick-borne encephalitis serocomplex flaviviruses from mosquito-borne flaviviruses. J Clin Microbiol. 2004;42:4101–10. 10.1128/JCM.42.9.4101-4110.2004 15364996 PMC516340

[7] Niedrig M, Vaisviliene D, Teichmann A, Klockmann U, Biel SS. Comparison of six different commercial IgG-ELISA kits for the detection of TBEV-antibodies. J Clin Virol. 2001;20:179–82. 10.1016/S1386-6532(00)00178-5 11166668

[8] Puchhammer-Stöckl E, Kunz C, Mandl CW, Heinz FX. Identification of tick-borne encephalitis virus ribonucleic acid in tick suspensions and in clinical specimens by a reverse transcription–nested polymerase chain reaction assay. Clin Diagn Virol. 1995;4:321–6. 10.1016/0928-0197(95)00022-4 15566853

[9] Ternovoi VA, Kurzhukov GP, Sokolov YV, Ivanov GY, Ivanisenko VA, Loktev AV, Tick-borne encephalitis with hemorrhagic syndrome, Novosibirsk region, Russia, 1999. Emerg Infect Dis. 2003;9:743–6. 12781020 10.3201/eid0906.030007 PMC3000154

[10] Ecker M, Allison SL, Meixner T, Heinz FX. Sequence analysis and genetic classification of tick-borne encephalitis viruses from Europe and Asia. J Gen Virol. 1999;80:179–85. 9934700 10.1099/0022-1317-80-1-179

[11] Bakhvalova VN, Potapova OF, Panov VV, Morozova OV. Vertical transmission of tick-borne encephalitis virus between generations of adapted reservoir small rodents. Virus Res. 2009;140:172–8. 10.1016/j.virusres.2008.12.001 19111585

[12] Labuda M, Nuttall PA, Kozuch O, Eleckova E, Williams T, Zuffova E, Non-viraemic transmission of tick-borne encephalitis virus: a mechanism for arbovirus survival in nature. Experientia. 1993;49:802–5. 10.1007/BF01923553 8405306

[13] Randolph SE, Green RM, Peacey MF, Rogers DJ. Seasonal synchrony: the key to tick-borne encephalitis foci identified by satellite data. Parasitology. 2000;121:15–23. 10.1017/S0031182099006083 11085221

[14] Phillips CJ. Harrington AM., Yates TL, Simpson GL, Baker RJ. Global disease surveillance, emergent disease preparedness, and national security. Lubbock (TX): Museum of Texas Tech University; 2009.

